# Complicated Management of Left-Sided Loculated Empyema Secondary to Streptococcus intermedius and Prevotella in a 53-Year-Old Male

**DOI:** 10.7759/cureus.47443

**Published:** 2023-10-21

**Authors:** Genevieve M Palmer, Khaled K Abdel Aziz, Amna Amir, Saba Waseem

**Affiliations:** 1 Internal Medicine, Conemaugh Memorial Medical Center, Johnstown, USA; 2 Emergency Medicine, Lake Erie College of Osteopathic Medicine, Bradenton, USA; 3 Internal Medicine, Lake Erie College of Osteopathic Medicine, Greensburg, USA

**Keywords:** loculated pleural effusions, intrapleural fibrinolytic therapy, video-assisted thoracic surgery (vats), thoracic empyema, pyothorax

## Abstract

We present the case of a 53-year-old male with complicated left-sided parapneumonic effusion due to *Streptococcus intermedius* and *Prevotella buccae*. Management required video-assisted thoracoscopic surgery and partial decortication of the left lung. Complications during the hospital stay were extensive, including sepsis, acute hypoxic respiratory failure, alcohol withdrawal, and transient ischemic attack.

## Introduction

Aspiration pneumonia is a feared complication in individuals with a history of alcohol abuse, owing to diminished cough reflex, impaired ability to clear noxious pathogens, and the presence of more pathogenic organisms such as *Klebsiella pneumoniae* [1]. Chronic alcohol use has additionally been found to be an independent predictor for increased risk of complicated parapneumonic effusion and empyema [2].

Parapneumonic effusion is defined as an accumulation of fluid in the pleural space as a result of pulmonary infection. Based on thoracentesis results, parapneumonic effusion is categorized as either uncomplicated effusion, complicated effusion, or empyema. Across all three subtypes, morbidity and mortality rates are high, particularly among elderly and immunocompromised patients [3]. Mortality for patients with empyema reaches as high as 20%, and the need for eventual surgery is a similar 20% [4]. Most commonly, parapneumonic effusion is addressed through non-surgical management, but in rare cases, surgical intervention may be warranted [5,6]. In this novel report, we present a patient with left-sided empyema that required both preoperative and postoperative intrapleural fibrinolytic therapy along with thoracoscopic surgery.

## Case presentation

This patient is a 53-year-old male who presented to the emergency department with a persistent, productive cough from three weeks duration. Significant medical history includes insulin-dependent diabetes mellitus, chronic kidney disease not requiring dialysis, schizophrenia, depression, hypertension, as well as alcohol abuse.

**Figure 1:**
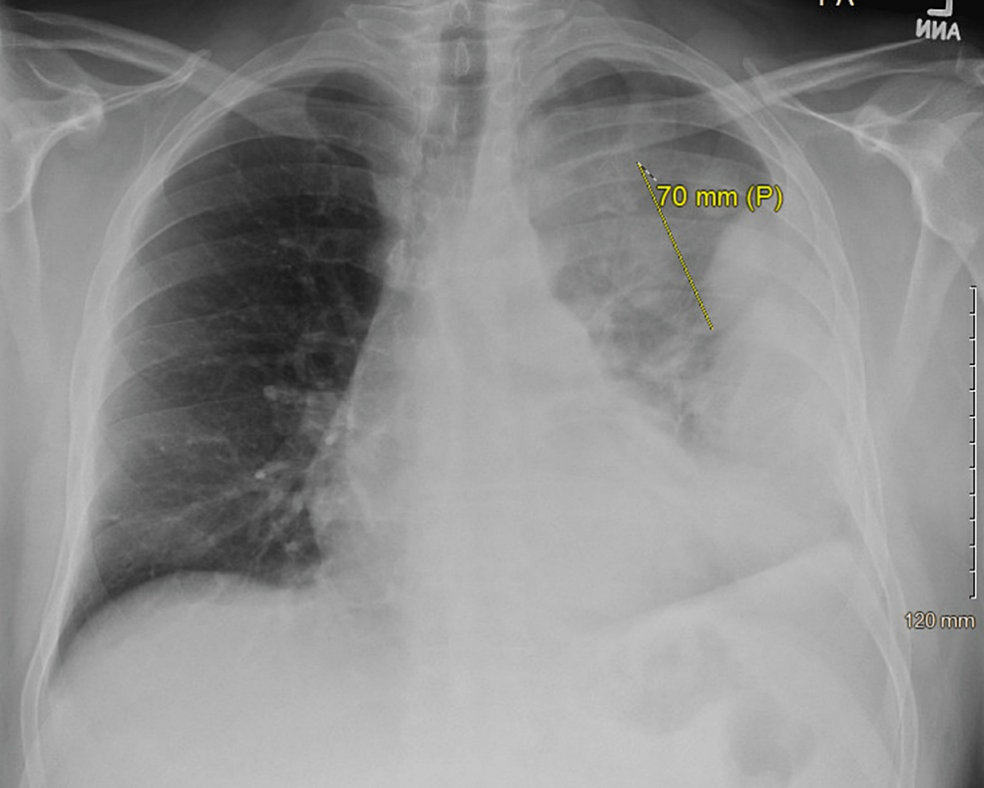
Chest radiograph obtained on admission demonstrating left upper lobe mass.

**Figure 2:**
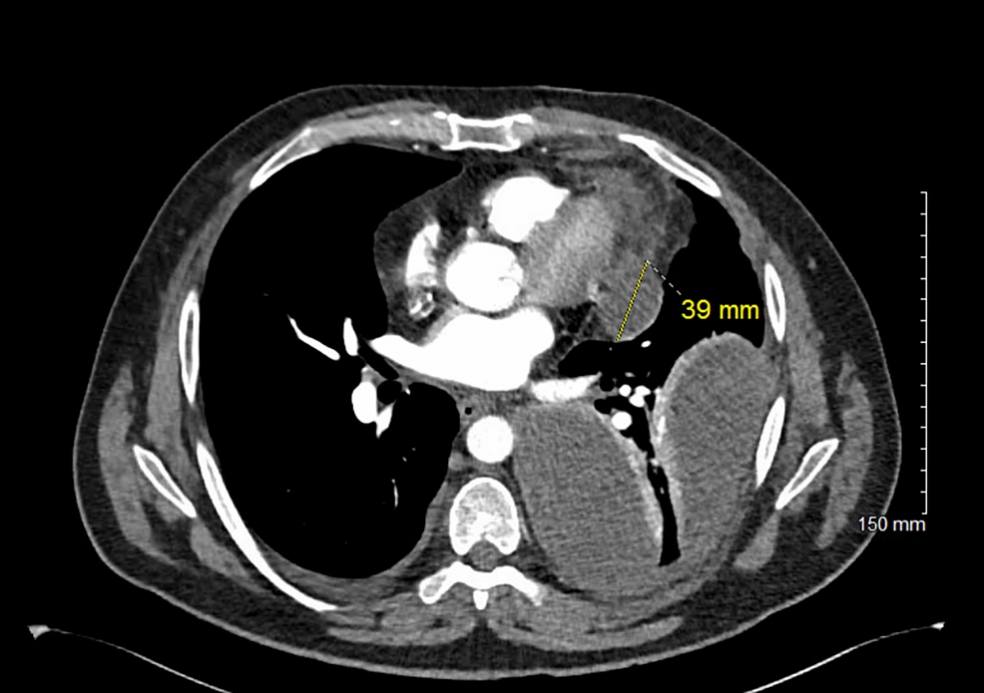
CT Pulmonary angiography axial view of multiloculated pleural effusion.

**Figure 3:**
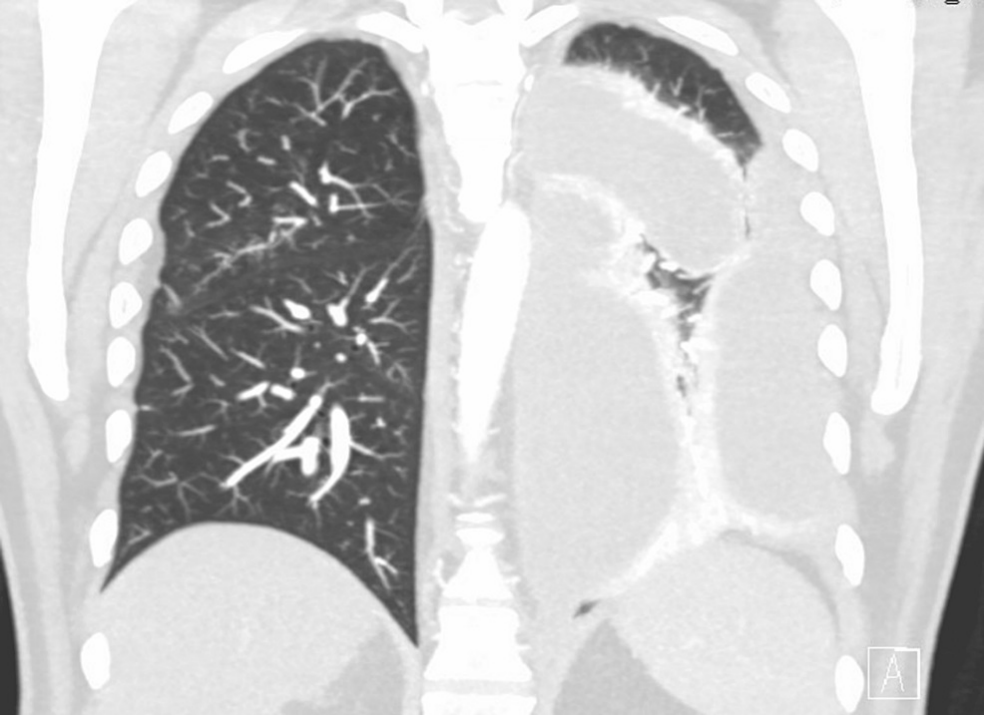
CT pulmonary angiography coronal view of multiloculated left pleural effusion.

Initial chest x-ray revealed a large left upper lobe mass with extensive lobular density laterally in the left hemithorax (Figure 1). CT pulmonary angiography subsequently demonstrated large multiloculated pleural effusion (Figures 2, 3). Significant laboratory findings included a sodium of 127 mmol/L (corrected for glucose), glucose 389 mg/dL, and elevated WBC 20.7 × 10^3^/µL with absolute neutrophils of 17.9 × 10^3^/µL. Antibiotic therapy was initiated with 1 g of ceftriaxone given via peripheral IV route as well as azithromycin 500 mg. The patient was later transitioned to 2 g IV cefepime, 2 g IV vancomycin, and 500 mg IV metronidazole.

 

The patient underwent left-sided 16F chest tube placement via blunt dissection surgical technique on day 2. Pleural fluid analysis demonstrated pH of 7.00, elevated white blood cells of 8,120 × 10^3^/µL with neutrophils at 89%, and elevated LDH of 1,433 U/L. Pleural fluid culture was positive for *Streptococcus intermedius* and *Prevotella buccae*. Cefepime, vancomycin, and metronidazole were continued, and WBC count decreased to 13.72 × 10^3^/µL by the third day of admission. The chest tube yielded more than 1,300 CC of pleural fluid and was replaced on day 5 of admission due to leakage. Antibiotics were switched to 3 g IV ampicillin sulbactam and 900 mg IV clindamycin.

**Figure 4:**
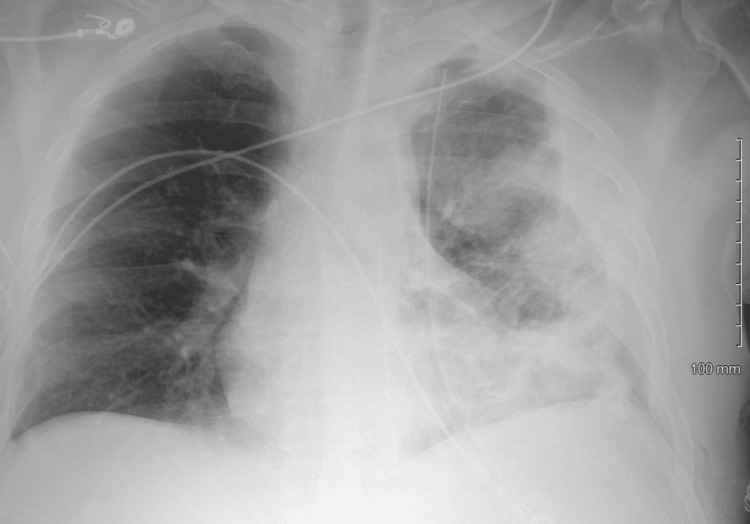
Chest x-ray obtained postoperatively.

Due to insufficient drainage through the second chest tube, as well as lack of clinical response to IV Unasyn, surgical intervention was recommended by cardiothoracic surgery. Patient underwent video assisted thoracoscopic surgery on day 12 which revealed inflamed and edematous pleura, purulence, and chronic inflammation. Partial decortication was performed, and two chest tubes were placed to ensure continued drainage. Two units of PRBC were given intra-operatively due to observation of blood in the thoracic space. Post-operatively, the patient required 6L O2 NC. A post-op chest x-ray is shown in Figure 4.

Following intrapleural instillation of 10 mg tissue plasminogen activator (tPA) and 5 mg deoxyribonuclease (DNase) via chest tube on POD 2, hemoglobin decreased to 7.1 g/dL and the patient was administered 1 unit of PRBC. Hemoglobin on POD 3 dropped again to 6.8 g/dL and the patient subsequently received 1 unit of PRBC. Hemoglobin was stable at 8.4 g/dL at discharge.

**Figure 5:**
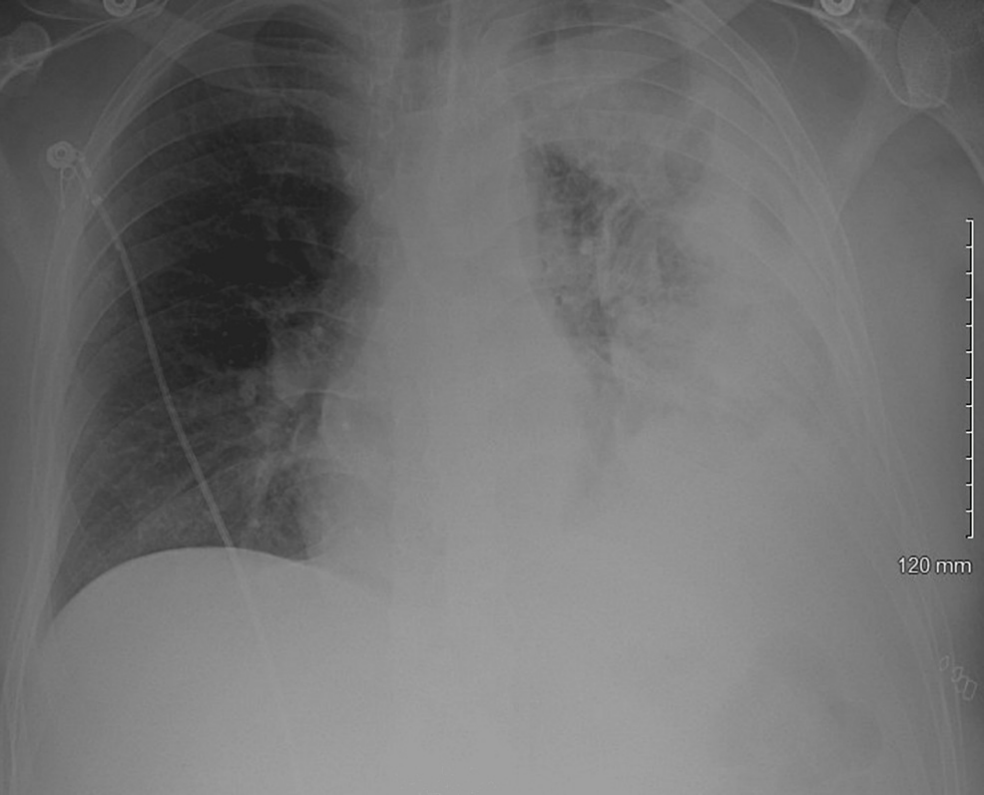
Chest x-ray obtained on the day of discharge.

Final chest x-ray revealed remaining alveolar and ground glass opacification of the left hemithorax as well as lenticular densities in the left hemithorax (Figure 5). The patient was discharged on hospital day 19.

## Discussion

With all three categories of parapneumonic effusion, appropriate antibiotic therapy is recommended [5]. Due to the risk factors of our patient in particular, the suspicion for aspiration pneumonia was high and thus coverage for oropharyngeal anaerobes was essential. Treatment with third-generation cephalosporins and metronidazole or treatment with beta-lactam and paired beta-lactamase inhibitor is recommended for this presentation in particular [7].

The decision to pursue operative management of empyema is complex and involves consideration of patient preference, comorbidities portending poor postoperative outcome, and source of infection. For patients with initial treatment of parapneumonic effusion and empyema with tube thoracostomy, loculation was found to be an independent predictor of failure of drainage [8]. The failure rate of intrapleural fibrinolytic therapy with tPA and DNase is estimated at 30% [9]. Early surgical intervention, particularly in patients with loculations/organized empyema, could result in a shorter length of stay and decreased need for open decortication of lung tissue [9].

Additionally, our patient developed an intrapleural hemorrhage after the administration of intrapleural tPA to treat complicated parapneumonic effusion. There is a need for further investigations to evaluate the safety and efficacy of intrapleural thrombolytics for the treatment of complicated pleural effusions and empyema.

## Conclusions

Management of complicated left-sided parapneumonic effusion, particularly with multiple loculations, is likely to require multiple methods of intervention. For patients with multiple loculations and complicated effusions, early consideration of surgical intervention could improve long-term patient outcomes. Finally, although further evaluation and study are needed, consider the risk of significant bleeding with the use of intrapleural thrombolytics when used for complicated effusion and empyema.
